# Common Whitethroats *Curruca communis* show a continuum of residency duration but a high degree of between‐years site fidelity at nonbreeding grounds in Nigeria

**DOI:** 10.1002/ece3.9334

**Published:** 2022-09-20

**Authors:** Claudia Tapia‐Harris, Will Cresswell

**Affiliations:** ^1^ Centre for Biological Diversity, School of Biology University of St Andrews St Andrews Scotland; ^2^ University of St Andrews A.P. Leventis Ornithological Research Institute Jos Nigeria

**Keywords:** Afro‐Palearctic migrant, common Whitethroat, *Curruca communis*, nonbreeding period, residency, return rates, site fidelity

## Abstract

The nonbreeding period represents a significant part of an Afro‐Palearctic migratory bird's annual cycle. Decisions such as whether to remain at a single site and whether to return to it across years have important effects on aspects such as survival, future breeding success, migratory connectivity, and conservation. During this study, we color‐ringed 337 common Whitethroats *Curruca communis* and undertook daily resightings to understand site persistence and the degree of site fidelity throughout three nonbreeding periods (November–April) in Nigeria. The probability of detecting a color‐ringed Whitethroat when it was present was 0.33. Site persistence varied widely across individuals (1–165 days) and did not differ significantly with sex or year, though first‐year birds remained for significantly shorter periods than adults. We believe that shorter residencies are likely due to the use of multiple stationary nonbreeding sites rather than low winter survival. A minimum of 19% of individuals returned to the study site the following year and shifted, on average, 300 m, suggesting that Whitethroats have a relatively high degree of between‐years site fidelity at a very fine scale. An individual's previous residency duration did not seem to determine its residency duration the following year. We suggest that spatial fidelity is high and constant through years, but temporal fidelity is not, and individual residency patterns vary, probably according to yearly and seasonal conditions. Our results highlight the complexity of the annual cycle of a single species and the importance of carrying out in situ, fine‐scale research throughout a migrant's annual cycle over several years.

## Graphical abstract

We colour‐ringed 337 Common Whitethroats *Curruca communis* and undertook daily resightings to study the winter residency, return rates, and degree of between‐years site fidelity throughout three nonbreeding periods in Jos, Nigeria. We found that spatial fidelity is high and constant between years, but temporal fidelity is not, and individual residency patterns vary, most likely according to yearly and seasonal conditions. In other words, timing is important: individuals revisit locations at very precise scales but not necessarily at the same time.

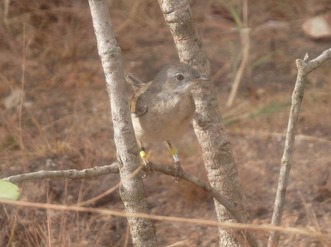

## 1 INTRODUCTION

Over 60% of an Afro‐Palearctic migrant's annual cycle occurs at nonbreeding grounds (McKinnon et al., 2013), where migrants experience unstable and challenging environmental conditions. What ensues during this period will have significant carryover effects on many aspects of their survival and reproduction (Both et al., 2006; Pulido, 2007) and on the overall population dynamics of a species. Nevertheless, this period has been insufficiently studied (Marra et al., 2015) and details regarding fine‐scale spatio‐temporal movements are lacking. Understanding site persistence, or residency, and the degree of between‐years site fidelity will contribute to a better understanding of migratory connectivity and of how birds may respond to longer‐term habitat and climate changes that, in turn, can lead to appropriate conservation efforts (Sanderson et al., 2006).

For many years, there was a largely evidence‐free assumption that small migrants tended to move across Africa, tracking changing seasonal conditions in a generally itinerant way (Moreau, 1972). More recently, there has been increasing evidence that this is strongly species‐ and population‐specific (Bulluck et al., 2019), with some species visiting several sites, others spending longer periods at fewer sites, establishing and defending territories, and, in some cases, showing both strategies (Belda et al., 2007; Blackburn & Cresswell, 2016; Catry et al., 2003; Thorup et al., 2019). To remain at a single site and maintain a territory confers advantages regarding local knowledge such as foraging locations, competitor densities, resource fluctuations, and predators (Catry et al., 2004; Piper, 2011), and avoids high costs and unpredictability associated with moving long distances, likely leading to higher survival rates (Cresswell, 2014; Yoder et al., 2004). On the other hand, itinerant individuals track ephemeral resources over a large area and are likely to move as environmental conditions change with the progression of the season, to optimize food availability (Ruiz‐Gutierrez et al., 2016).

Many Afro‐Palearctic migrants not only remain for prolonged periods at nonbreeding sites but return to them year after year, especially territorial individuals (Barshep et al., 2012; Blackburn & Cresswell, 2016; Cuadrado, 1992). Familiarity with these sites confers similar advantages as longer residency. Furthermore, fidelity has also been detected at a temporal scale, where individuals return to the same sites during similar times of the year (Stanley et al., 2012; van Wijk et al., 2016).

According to the serial residency hypothesis (Cresswell, 2014), many Afro‐Palearctic migrants are likely to be faithful to any site(s) that promotes their survival, thus we expect strong residency differences and return rates among individuals of different ages. This hypothesis predicts that first‐years, which lack knowledge of small‐ and medium‐scale locations of where to arrive, will reach the nonbreeding grounds stochastically. Some will find a site and remain at it until migration, while others will continue their search elsewhere, many of them arriving at less suitable sites or even discovering new unknown suitable habitats. Individuals will then reuse those successful sites during subsequent years as adults. Therefore, if an individual gets older, it becomes more site faithful because of natural selection removing those that did not locate suitable sites. In any population, therefore, older birds will be more site faithful.

Studying site persistence and between‐years site fidelity, however, is problematic. First, few species are likely to be so noticeable that individuals will always be detected at a site when present, leading to false negatives, particularly with low sampling effort. Second, determining site persistence and return rates greatly depends on when individuals are first marked and on their duration of stay, because passage birds will have lower detection and overall capture probabilities than more resident birds. Third, data are highly dependent on the methods used (e.g., ringing schemes, geolocators, resightings). Ringing studies, for example, are usually undertaken at the beginning and end of the season at constant sites. This increases a bird's “net‐shyness,” reduces capture probability, and makes it difficult to detect short‐duration stays. Results from year‐round tracking studies, on the other hand, are potentially the solution, except that small passerines are commonly tracked with archival tags, where data are only recovered if an individual has some degree of site fidelity and at a very low spatial resolution, or they are tracked with radio tags, which cannot be detected over their entire range.

**FIGURE 1:**
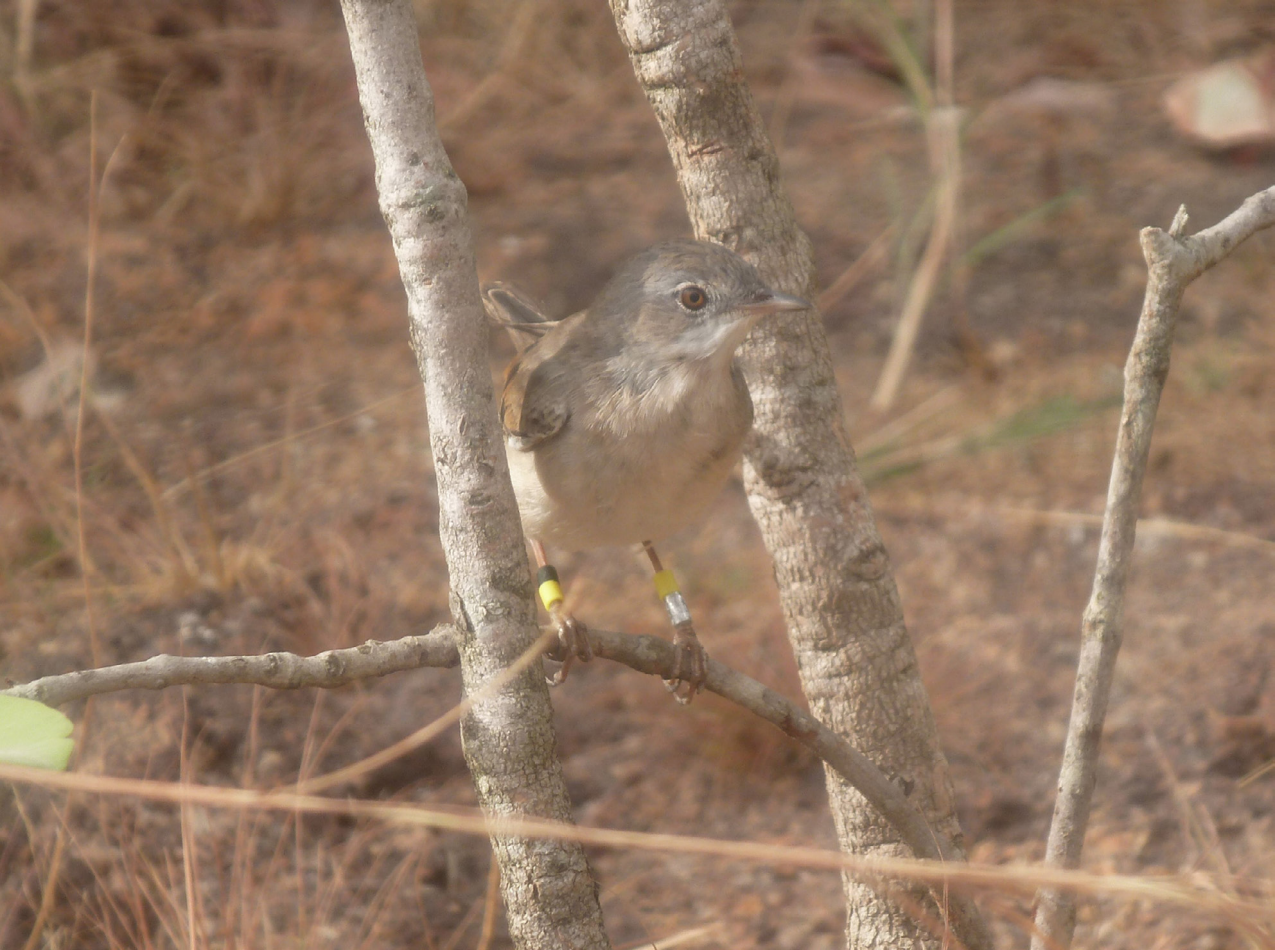
Adult Common Whitethroat color‐ringed in Nigeria. This individual was ringed on 23 March 2019 at 8:18 am. There were no subsequent detections. Photograph: Claudia Tapia‐Harris.

The Common Whitethroat *Curruca communis* (henceforth “Whitethroat”; Figure 1) is a widely distributed small Afro‐Palearctic migrant. Studies based on ringing recoveries and sporadic encounters have speculated that Whitethroats are faithful to their nonbreeding grounds and remain there for a considerable period. Whitethroats inhabit dense thickets, show inconspicuous behavior, and are relatively quiet during the nonbreeding period (Zwarts & Bijlsma, 2015), all of which make them hard to detect. Despite this, Whitethroats seem to be a typical long‐distance Afro‐Palearctic passerine migrant that can provide useful information applicable to other migrants. In this study, we use intensive resighting efforts throughout three nonbreeding periods at a single site in Nigeria to understand, at an individual level, how Whitethroats use the nonbreeding grounds at a fine spatial and temporal scale. We first calculate the probability of detecting an individual during a resighting visit and investigate the site persistence of individuals of different age and sex groups. We then describe return rates and the degree of between‐years site fidelity and determine whether individuals depart the area at similar times every year. To our knowledge, this is the first study to research winter residency and site fidelity of Whitethroats at a very fine spatial scale during the entirety of multiple nonbreeding seasons. Note that throughout this manuscript winter refers to winter in the Northern Hemisphere.

## 2 METHODS

### 2.1 Study site

The study took place over three consecutive nonbreeding periods, hereby referred to as year 1 (November 2017–April 2018), year 2 (September 2018–April 2019), and year 3 (November 2019–March 2020) at a guinea savannah site on the Jos Plateau, Nigeria in West Africa (09°52′N, 08°58′E). This region experiences single pronounced wet and dry seasons lasting 6 months each, from May to October and November to April, respectively. Sites were primarily open scrubland with different and varying degrees of anthropogenic activities, e.g., farming, livestock grazing, tin mining, and fires (Hulme & Cresswell, 2012). These sites represent typical African dynamic habitats, where anthropogenic activities are constant and continuously changing throughout the year.

### 2.2 Mist‐netting and resightings

Birds were captured using 9 m, 12 m, and 18 m × 2.5 m 5‐shelf (16 × 16 mm mesh) mist nets and conspecific playback. During year 1, nets were set up in the morning between mid‐November 2017 and mid‐February 2018 (mean of four nets per day, open for 2 h 50 m), totalling 70 visits. In year 2 nets were set up in the morning and/or evening from late October 2018 to mid‐April 2019 (mean of 4.5 nets per day for 3 h 24 m), totalling 69 visits. A few additional birds were caught in year 3 between mid‐November 2019 and mid‐February 2020 but were excluded from return rates and between‐years site fidelity analyses because they were not sought out the following nonbreeding season. All individuals were sexed as either female, male, or unknown, and aged as either first‐year, adult, or unknown (Svensson, 1992). Each individual was given a unique combination of colored leg rings (three color rings and a metal ring). In total, 212 individuals were color‐ringed in year 1, 115 individuals in year 2, and 10 in year 3. This work was conducted under the ethical guidelines of the A.P. Leventis Ornithological Research Institute Scientific Committee and all methods were approved by the School of Biology Ethics Committee of the University of St. Andrews (SEC17028).

Resightings were carried out at least once a week between sunrise and ~1030 h and/or between ~1500 h and sunset throughout the fieldwork period. Two observers undertook all observations. We interspersed starting points to avoid biases as a product of the time of day and air temperature. Resightings were not carried out during days of heavy rain. Once an individual was detected we proceeded to identify its complete color combination using 10 × 40 binoculars. GPS points were recorded with a Garmin eTrex10™ GPS where individuals were first detected and/or captured. Due to the skittish and shy behavior of Whitethroats, conspecific playback was used. In some cases, individuals were first detected and playback was then used to help reveal the complete color combination. In most cases, however, when there were no signs of activity, playback was used before detection. This did not seem to induce any significant movement in individuals, and we believe that most recorded GPS points reflect unbiased locations where the individuals would be without any interaction with observers. We tried to spend the same effort resighting all individuals, but we acknowledge that this may not have always been the case. 135 individuals were seen at least once after capture. Because of the high resighting effort, we are confident that departure months and site persistence were determined accurately.

### 2.3 Radio tag deployment

Between 25 October and 28 November 2018, 11 individuals were fitted with “LifeTags™,” a 0.45 g solar‐powered and battery‐free radio transmitter from Cellular Tracking Technologies™. Tags were attached to birds' backs using an elastic leg‐loop harness (Rappole & Tipton, 1991). Devices weighed approximately 0.51 g with the harness, corresponding to 3.4% (3.2%–3.8%) of an individual's body mass. As individuals were fitted with radio tags, an effort was made to seek them at least twice a week after tag deployment until 8 December 2019. All birds were observed for at least 3 days after tag deployment. When individuals were detected, efforts were made to observe and corroborate the ring combination. GPS coordinates were recorded where individuals were first seen or heard or when detection was strong. To determine whether radio tags had any negative effect on individuals, the residency period (number of days between when an individual was caught and the last time it was detected) and return rates (proportion of individuals that returned the following nonbreeding period) were compared between 11 radio‐tagged individuals and 11 randomly selected control birds, ringed during the same period. No significant differences were found regarding residency periods (*F*_[1,20]_ = 0.05, *p* = .82) or return rates (χ^2^ = 0.26, df = 1, *p* = .61) between radio‐tagged individuals and controls.

### 2.4 Statistical analyses

All data were analyzed using R version 3.6.3 and RStudio version 1.1.456 (R Core Team, 2020), and a statistical significance level of *p* < .05 was chosen to reject the null hypotheses.

#### 2.4.1 Detection probabilities

The probability of detecting an individual directly affects how we calculate and categorize site persistence. Therefore, to estimate detection probability we used multiple data sets and methods.

1. *Manually*: Detection probabilities were calculated by dividing the number of times a bird was detected (number of encounters) by the total visits to its home range between its first detection (excluding the date it was ringed) until its last detection for each year. We used data obtained from individuals that we knew were present at the study site during each visit (i.e., obvious long‐term winter residents, see below) to be certain that their nondetection was due to detectability factors and not due to absence or death. This assumes that birds did not leave their home range at any time and that all birds, if present, had the same probability of detection. We used information collected from 20, 16, and 15 individuals during years 1, 2, and 3, respectively. All data were analyzed separately by year and returning individuals were included in every year they were detected: excluding them would otherwise bias estimates by preferentially sampling first‐year birds.
2. *MARK*: With the same data, we proceeded to calculate detection probabilities using Cormack–Jolly–Seber (CJS) models in MARK software (White & Burnham, 2009). CJS models estimate both apparent survival (*φ*) and detection probability (*p*), where the former is the probability that an individual survives from one sampling occasion to the next, and the latter is the probability that, given that the individual is alive and in the sample, it is encountered (Hammond, 2018). Given that we used capture histories from individuals who we knew were present and alive (*φ* = 1), we were only interested in obtaining the detection probability for each year. We assumed that detection probability was constant throughout all encounters (*φ*(.)*p*(.)).
3. *Radio tags*: Detection probabilities were calculated for three radio‐tagged individuals that were detected during at least three visits in year 2. Every time a radio‐tagged individual was detected with the antenna, we proceeded to find it in the same manner that we would normally do during resightings. We then estimated detection probabilities by dividing the number of visits during which an individual was detected in “resighting” conditions by the total number of visits that the same individual was detected with the radio tag antenna.

The final overall detection probability was obtained by averaging all seven estimates: probabilities obtained manually and in MARK for all three seasons (total of six probabilities), and a probability obtained through radio‐tagged individuals. Additionally, to compare whether the mean detection probability was constant between nonbreeding periods and methods, General Linear Models (GLMs) were performed.

#### 2.4.2 Site persistence

Once established that individuals undertook different residency strategies (see Appendix A), we estimated the number of days individuals spent in the study area (days between when individuals were first and last detected). To facilitate further comparisons, however, individuals were grouped into residency categories as seen in Table 1. Individuals detected across more than 1 year were categorized independently each year.

**TABLE 1:** Description of residency categories and the number of individuals within each category per year. Percentages, excluding unknown birds, are shown in parenthesis

Residency category	Description	Year 1	Year 2	Year 3
Long‐term winter residents	Remained >=60 days at the study site, was detected two or more times after ringing, and seen at least once after January.	41 (30%)	31 (35%)	21 (52%)
Short‐term winter residents	Remained between 8 and 59 days at the study site.^a^	7 (5%)	15 (17%)	7 (18%)
Passage birds	Ringed between October and December. Only detected when ringed and remained ≤7 days at the study site.	90 (65%)	43 (48%)	12 (30%)
Unknown	Ringed between January and April. Only detected when it was ringed, or pattern was not clear.	*44*	*55*	*0*
	Total	138 (182)	89 (144)	40 (40)

*Note*: All categories could include an unknown number of individuals that may not have migrated beyond our study site but could have settled close by and gone undetected. Note that these categories do not differentiate between departures and mortality.

a If individuals were ringed during January or February, they could potentially be long‐term winter residents.

We performed GLMs to understand whether site persistence, defined as the number of days an individual was present and detected in the area, varied across years, age, and sex. Birds that could not be aged or sexed were excluded from models that included these variables as predictors. First‐year Whitethroats are difficult to sex accurately (Waldenström & Ottosson, 2000), so models using sex as an independent variable only include adults. Because of this, modeling for the effects of age and sex in residency periods was undertaken separately. Data from year 3 were excluded from these analyses and those individuals whose age and sex were unknown.

We used a model averaging approach for models that had the same sample size using the “*dredge*” and “*model.avg*” functions from the “*MuMin*” package in R (Barton, 2020). This procedure entails carrying out all possible models from a base model (i.e., “days ~ age + year” and “days ~ sex + year”) and calculating a weighted average of parameter estimates, such that parameter estimates from models that contribute little information about the variance in the response variable are given little weight (Grueber et al., 2011).

#### 2.4.3 Between‐years site fidelity

Return rates were estimated by dividing the number of individuals that were seen in year *i* + 1 by the total number of individuals ringed in year *i*. To determine the degree of between‐years site fidelity of individuals that returned for at least two nonbreeding seasons—how far an individual moved from year *i* to year *i* + 1—we calculated the centroid coordinate for each individual in each year and estimated the distance between centroids using the “*distHaversine*” function from the “*geosphere*” package version 1.5.10 in R (Hijmans, 2019). Individuals were grouped into group A, individuals detected in years 1 and 2, group B, individuals detected in years 2 and 3, and group C, individuals detected in years 1 and 3 but not in year 2. Individuals that were seen during all three seasons were not excluded from the analysis and were added to groups A and B.

Chi‐squared tests (χ^2^) were performed to determine the effects of year, age at year *i* (“previous age”), sex, and residency at year *i* (“previous residency”) on return rates. A model averaging approach was also undertaken to explore whether the distance moved from 1 year to another was dependent on previous age, sex, and previous residency (base model: “dist ~ preage + sex + group + preres + preage × preres + preage × group”). All birds that could not be aged were excluded from models that included age as a predictor.

#### 2.4.4 Residency repeatability

To explore whether individuals remained for similar periods across different years, or whether they repeated residency categories the following years, we estimated the percentage of individuals that remained (or changed) in each residency category. We carried out a linear model and estimated the correlation between the number of days spent in year *i* with the number of days spent in year *i* + 1.

#### 2.4.5 Departure dates

We tested the departure date repeatability of individuals seen for at least two nonbreeding periods. Year 3 birds were excluded from this analysis because resightings that year ended earlier, and final resightings were not likely to reflect true departure dates. We excluded records of all birds that were seen after 25 February (3 weeks before the end of observations) to exclude birds that were highly likely to have not left before our last resighting effort of that year. Note that departure from our study site does not necessarily imply the commencement of spring departure but could also reflect a movement to another nonbreeding site and should, thus, be treated with caution. We estimated repeatability using the “*rpt*” function in the “*rptR*” package (Stoffel et al., 2017). This uses a linear mixed model framework where the groups compared for repeatability are specified by a random effect (i.e., individuals). Confidence intervals were estimated by running 1000 bootstraps. We calculated repeatability for adults and first‐year birds, as well as for each residency category (i.e., long‐term, short‐term, and passage birds).

To describe population variation regarding departure dates, we pooled all observed dates across the first 2 years from individuals that left after January in a respective year. We then calculated the difference between each date and the date of earliest sighting and calculated the mean, standard error (SE), and range. To describe intra‐individual variation, we used data from individuals that were detected for at least 2 years. We calculated the difference between the two values for each individual observed in 2 years and calculated the mean, SE, minimum and maximum values across all individuals. GLMs were performed to test for differences between individuals categorized by previous residency and previous age.

## 3 RESULTS

### 3.1 Detection probabilities

**FIGURE 2:**
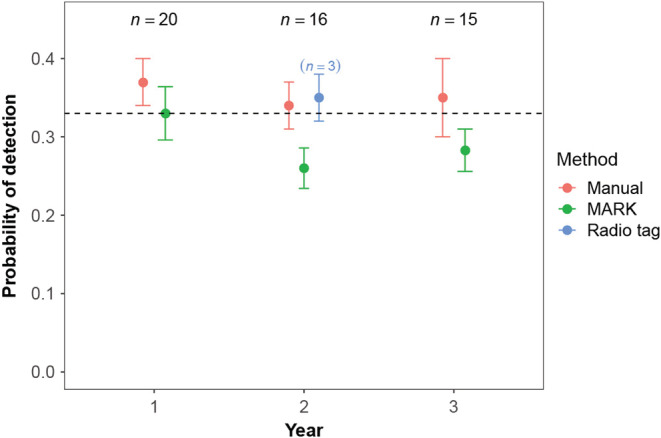
Mean detection probabilities ± 1 standard error of known long‐term resident birds in each year using distinct methods (red = manually, green = MARK, blue = manually from radio‐tagged birds). Sample sizes during each year are shown on the top. In year 2 “(*n* = 3)” represents the sample size of radio‐tagged birds. Dashed line shows the overall mean detection probability (.33). There were no clear differences in detection probabilities across years or methods.

The mean of all seven detection probabilities (manual probabilities from years 1, 2, and 3, MARK probabilities from years 1, 2, and 3, and probability from radio‐tagged birds in year 2) was 0.33 (SE = 0.02); the probability of detecting a Whitethroat at our study site when it is present was once every three visits (Figure 2). Detection probabilities were similar between years when undertaken manually (mean = 0.36, SE = 0.02, *F*_[2,48]_ = 0.13, *p* = .88) and in MARK (mean = 0.29, SE = 0.03, *F*_[2,48]_ = 1.48, *p* = .24) and were similar across methods during all 3 years (year 1: *t*(_38_) = 0.88, *p* = .38; year 2: *F*_(2,32)_ = 2.44, *p* = .10; year 3: *t*(_28_) = 1.18, *p* = .25) Figure 2.

### 3.2 Site persistence

Site persistence, or residency, defined as the number of days an individual was present and detected in the area, varied widely across individuals, ranging from 1 day to 165 days (mean = 31 days, SE = 3 days, *n* = 341) but did not seem to differ significantly between years or between adult female and male birds (Table 2). First‐years (mean = 29 days, SE = 4.5 days, *n* = 99), however, remained for significantly shorter periods when compared to adults (mean = 45 days, SE = 5 days, *n* = 105) (Table 2).

**TABLE 2:** General linear model results of site persistence and distance shifted between years predictors. In models, age = adult, sex = female, year = 1, group = a, residency = long‐term were the base categories. Significant *p* values are highlighted in bold and italics. All interactions were not statistically significant.

Site persistence				
Age *n* = 204				
Full model average (days ~ age + year)				
Variable	Estimate	Adjusted SE	*z*	*p*
(Intercept)	40.9	6.98	5.85	
Age first‐year	−15.25	7.03	2.16	**.03**
Year 2	10.17	7.39	1.36	.17
Sex *n* = 81				
Full model average (days ~ sex + year)				
Variable	Estimate	Adjusted SE	*z*	*p*
(Intercept)	48.43	8.86	5.47	
Year 2	10.75	12.04	0.89	.37
Sex Male	8.1	11.88	0.68	.5
Distance shifted between years				
*n* = 47				
Full model average (dist ~ preage + sex + group + preres + preage*preres + preage*group)				
Variable	Estimate	Adjusted SE	*z*	*p*
(Intercept)	345.30	153.49	2.25	
Group B	−243.58	182.12	1.34	.18
Group C	−235.04	482.60	0.49	.62
Preage First‐year	−135.23	186.66	0.72	.47
Group B: Preage First‐year	1975.10	391.74	5.04	**<.001**
Group C: Preage First‐year	528.83	592.42	0.89	.37
Preresidency Passage	180.12	152.54	1.18	.24
Preresidency Short‐term	2.69	228.09	0.01	.99
Sex Male	86.32	161.39	0.54	.59
Sex Unknown	215.19	182.53	1.18	.24
Preage First‐year: Preresidency Passage	40.20	309.05	0.13	.90
Preage First‐year: Preresidency Short‐term	−75.23	560.48	0.13	.89

### 3.3 Between‐years site fidelity

#### 3.3.1 Return rates

Overall return rates were similar across years but varied between age groups dependent on year and residency category, with more long‐term and short‐term winter residents returning than passage birds. A similar proportion of individuals returned between years (χ^2^ = 0.56, df = 1, *p* = .45): 36/182 (20%) individuals returned from year 1 to year 2 (group A), and 24/145 (17%) individuals returned from year 2 to year 3 (group B). Seven individuals from year 1 failed to return in year 2 but then returned in year 3 (group C). Only 12 individuals were seen during all three fieldwork seasons. In group A, a similar proportion of individuals of adults and first‐year birds returned the following year: 13/62 (21%) adults and 22/96 (23%) first‐years (χ^2^ = 0.08, df = 1, *p* = .77). In group B, however, there were clear differences between individuals of different ages: 20/90 (22%) adults and 3/50 (6%) first‐years returned (χ^2^ = 6.16, df = 1, *p* = .01). At least four individuals from group C were first‐year birds in year 1. Female and male adults had similar return rates in group A (females = 5/21, 24%, males = 6/28, 21%; χ^2^ = 0.04, df = 1, *p* = .84) and in group B (females = 9/35, 26%, males = 8/42, 19%; χ^2^ = 0.49, df = 1, *p* = .48). When comparing return rates among residency categories in group A, long‐term winter residents (14/43, 33%) and short‐term residents (2/7, 29%) had higher return rates than passage birds (10/90, 11%) (χ^2^ = 9.34, df = 2, *p* = .009). A similar trend was seen in group B (χ^2^ = 6.98, df = 2, *p* = .03); 12/31 (39%) of long‐term winter residents returned; 3/16 (19%) short‐term residents; and 5/40 (13%) passage birds.

**FIGURE 3:**
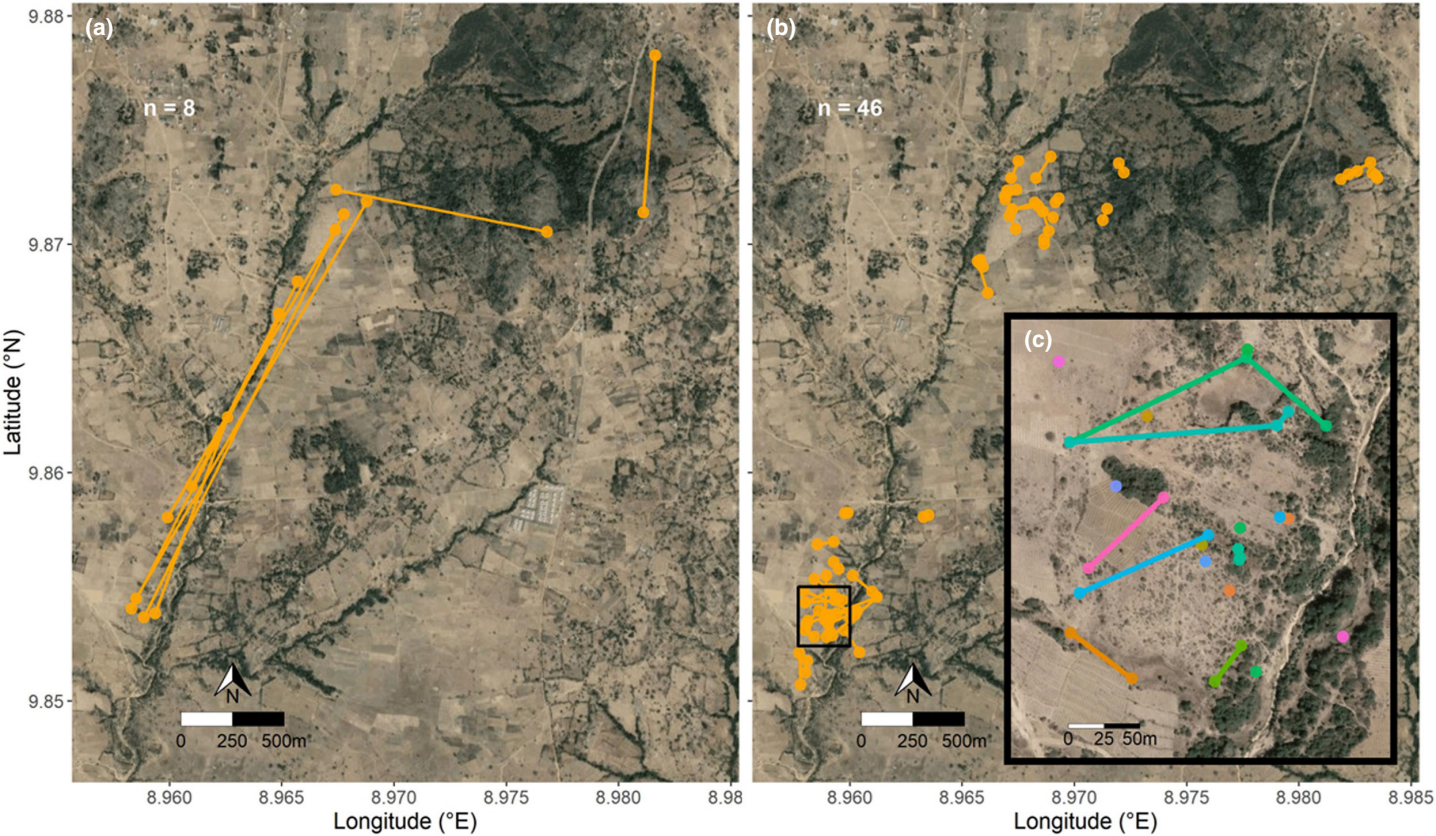
Distances moved between year *i* and year *i* + 1 by individuals that moved above the average (> 300 m; a) and below the average (< 300 m; b). Sample sizes are shown on each map. A subset of individuals is shown with a higher definition in map c). Here, each color represents a different individual. Individuals that do not have a line moved out of the confines of the box. Overall, individuals had a high degree of between‐years site fidelity.

The distance moved from 1 year to another varied among individuals (Figure 3) but, on average, individuals moved less than 300 meters (Figure 3; Appendix B). This figure was similar among groups A, B, and C (*F*_[2,51]_ = 0.006, *p* = .99).

**FIGURE 4:**
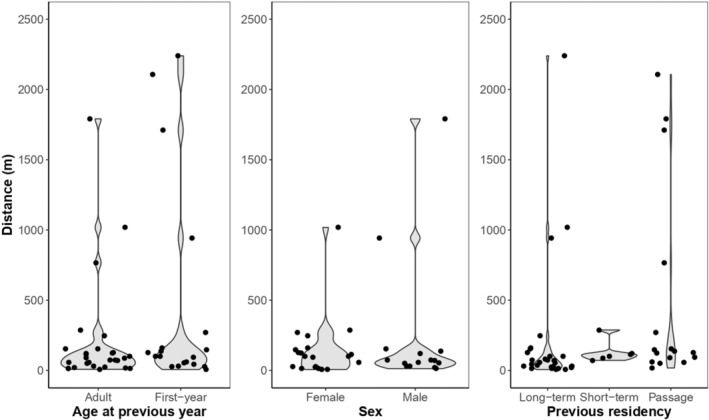
Distance (meters) moved from year *i* to year *i* + 1 according to previous age, sex, and previous residency.

The distance shifted between years did not vary significantly according to previous age (*F*_[1,45]_ = 2.1, *p* = .16), sex (*F*_[1,33]_ = 0.58, *p* = .45) or previous residency (*F*_[2,47]_ = 1.61, *p* = .21; Figure 4; Appendix B). Results from the averaging model, however, show that first‐years in group B (seen from year 2 to year 3) shifted longer distances than adults (Table 2). All other variables were not statistically significant (Table 2).

#### 3.3.2 Residency repeatability

**FIGURE 5:**
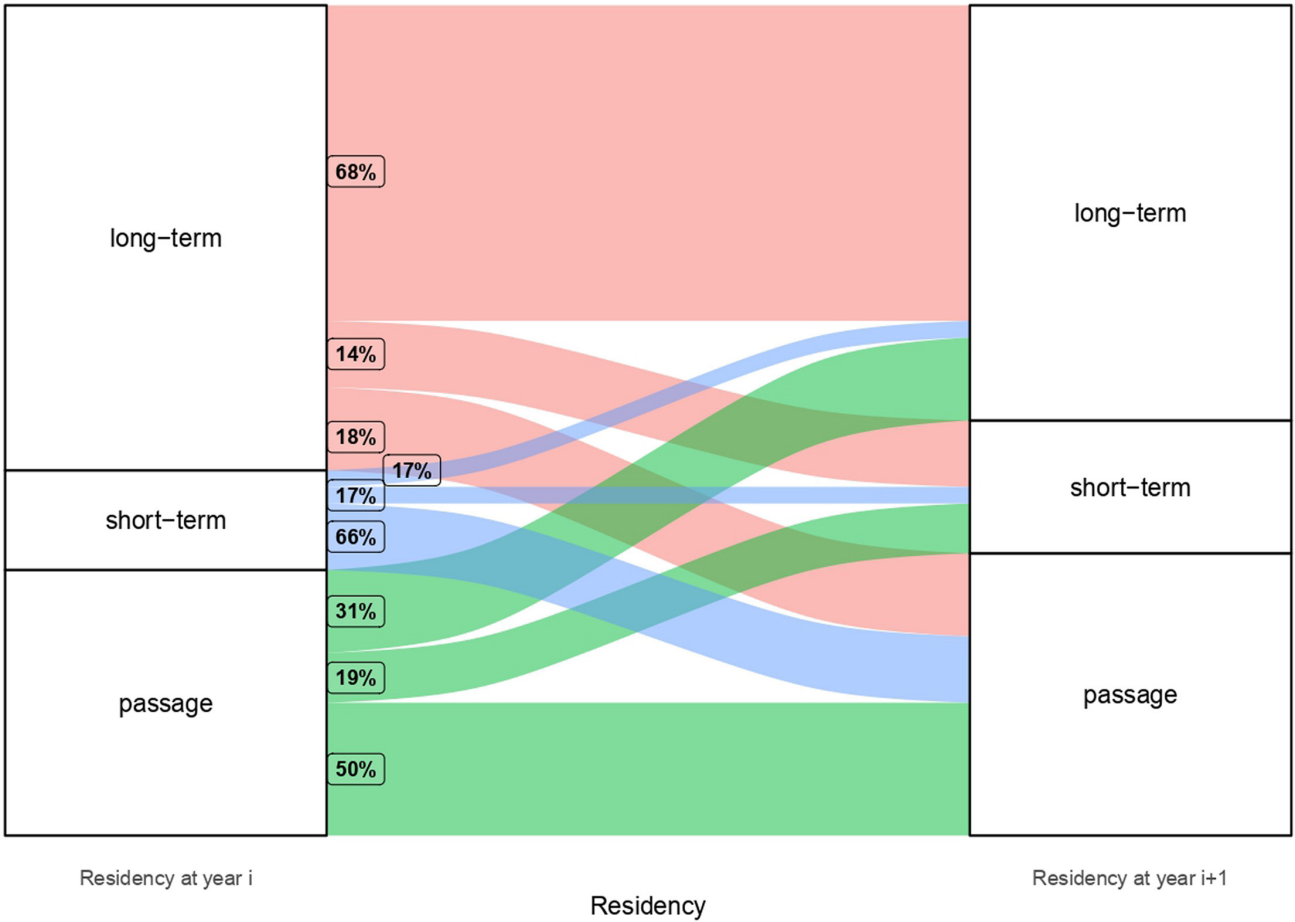
Change in individuals' residency category from 1 year to the following year it was detected. Percentage in the square represents the proportion of individuals in each residency category at year *i* + 1, as is observed by the width of the bands. Colors represent previous residency category.

The degree of residency category repeatability, i.e., whether individuals remained in the same residency category through different years, varied across individuals (Figure 5). 68% of long‐term winter residents remained as such the following year, and 32% remained for similar or shorter periods. Most of the short‐term winter residents (66%), when they returned the following year, were categorized as passage birds, although 17% remained for similar periods and 17% remained for longer. Half of the passage birds remained as such the following year, while the other half remained for longer periods: 31% were categorized as long‐term winter residents and 19% as short‐term winter residents (Figure 5). Additionally, half of the birds categorized as unknown were short‐term winter residents the following year, while the other half were long‐term winter residents.

**FIGURE 6:**
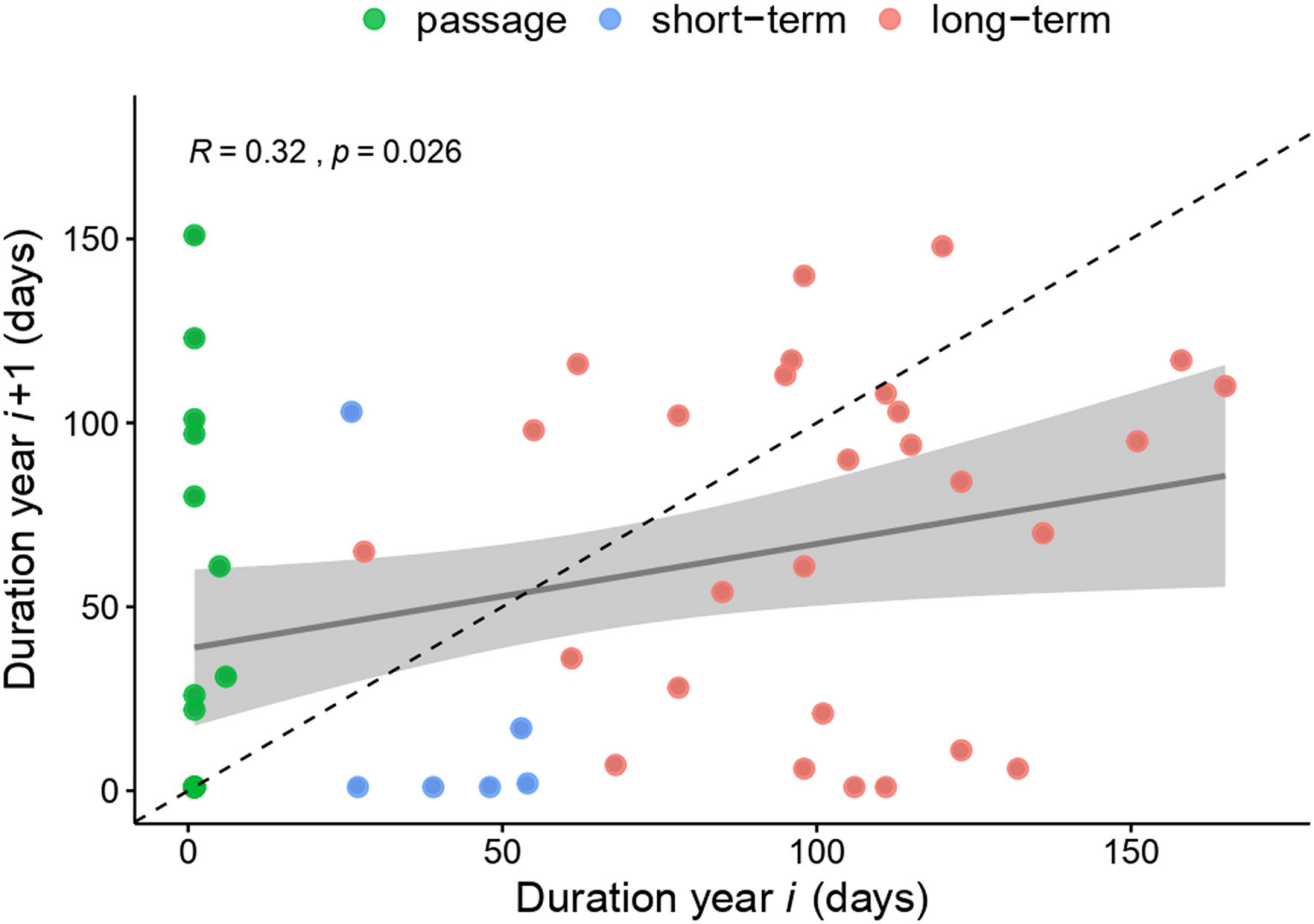
Linear correlation between the number of days an individual spent at the study site in year *i* and the number of days an individual spent at a location in year *i* + 1. Colors represent residency category during year *i*. The dotted line represents a constant residency period during both years. Points above the dotted line represent individuals that remained longer in year *i* + 1 than the previous year and points below the line represent individuals that remained a longer period in the previous year. *R* = correlation between variables and the *p*‐value shows a significant positive trend.

When comparing the duration (in days) spent at the site of individuals from 1 year to another, we found that there was a small but significant positive correlation between the duration in year *i* and the duration in year *i* + 1 (correlation *R* = .32, *p* = .026): individuals that remained for longer periods in year *i* remained for longer periods in year *i* + 1 but, overall, individuals remained for shorter periods the following year (Figure 6). The latter is especially true for short‐term and long‐term winter residents. Passage birds, however, remained for longer periods during year *i* + 1 compared with during year *i* (Figure 6).

### 3.4 Departure dates

Departure dates for individuals seen between January and April during years 1 and 2 did not vary between years (*F*_[1179]_ = 0.02, *p* = .90), between adults and first‐years (*F*_[1179]_ = 0.002, *p* = .89), or between males and females (*F*_[1137]_ = 0.03, *p* = .31). Individuals that were seen during at least 2 years showed relatively low repeatability values (*r* = .15, Table 3). The difference (in days) between the departure date in year *i* and that of year *i* + 1 was statistically significant when categorizing individuals by their residency at year *i* (*F*_[2,37]_ = 4.3, *p* = .02). This means that long‐term birds departed at more similar dates across years compared with passage birds (Table 3). When categorizing individuals by their previous age, we found that there was no significant difference in departure dates between adults and first‐year birds (*F*_[1,37]_ = 0.27, *p* = .61).

**TABLE 3:** Intra‐individual and population variation of departure dates (shown as ordinal days) of individuals seen in years 1 and 2 separated by age and residency category

		Intra‐individual variability (individuals seen during both years)								Population variability (all individuals from years 1 and 2)			
		*n*	Mean ± SE	Median	Min	Max	*r*	CI	*p*	*n*	Mean ± SE	Median	Max diff
Age	Adult	21	46 ± 8.8	39	1	126	.10	0–0.492	.37	104	62 ± 2.5	68	109
	First‐year	18	52 ± 8.6	55	4	120	.146	0–0.56	.31	80	62 ± 3	68.5	98
Residency	Long‐term	20	36 ± 8.4	20	4	126	.147	0–0.401	.16	72	57 ± 2.4	60	92
	Short‐term	6	33 ± 10	36	3	69	.10	0–0.494	.37	14	42 ± 8.6	39	89
	Passage	14	71 ± 10.3	77	1	124	.146	0–0.568	.31	11	48 ± 9.7	35	88

## 4 DISCUSSION

We researched site persistence and the degree of between‐years site fidelity of Whitethroats located within a small area in central Nigeria throughout three consecutive nonbreeding periods. We first estimated that the probability of detecting an individual when it was present, was 0.33. Site persistence varied widely across individuals, ranging from one to 165 days, and first‐year birds remained for significantly shorter periods than adults. A minimum of 19% of individuals returned to the study site the following year and shifted ~300 m. An individual's previous residency duration did not determine the residency duration the following year. Overall, the departure timing of individuals that were detected during late winter was similar across years, but long‐term birds departed at more similar dates across years compared with passage birds. At the end of this section, we will attempt to explain these results in a single modification to the serial residency hypothesis—strong location fidelity across years, but the timing of movement to additional sites determined by conditions in each year.

Prior to discussing these results, however, it is important to restate that direct comparisons between studies looking into residency and fidelity of long‐distance migrants at a fine scale, although important, are not ideal. Itineracy and residency are, in practical terms, subjective and depend greatly on the duration of the study, as well as how these terms are defined. Additionally, differences between studies, such as the methods used and when and at which part of the species' nonbreeding range they took place, make comparisons complicated.

### 4.1 Detection probabilities

The probability of detecting a color‐ringed Whitethroat at our study site, when it was present, was 33%. Our results are consistent with the *Sylviidae* family having relatively lower detection rates than other passerine birds (Johnston et al., 2014; Zwarts & Bijlsma, 2015) and are similar to detection probabilities at their breeding sites in the UK (30%; Johnston et al., 2014). This rate is relatively low when compared to detectability at the nonbreeding grounds of other Afro‐Palearctic migrants such as Whinchats *Saxicola rubetra* (63% detection probability; Blackburn & Cresswell, 2016) and Chiffchaffs *Phylloscopus collybita* (recapture probability 66%; Catry et al., 2003), though few studies have addressed and calculated detection probabilities during this period (e.g., Zwarts & Bijlsma, 2015). Nevertheless, despite Whitethroats having relatively low detection probabilities, we consider that our high sampling effort (sites were visited at least once a week, for over 20 weeks each year) was sufficient to compensate for this.

### 4.2 Site persistence

Site persistence varied significantly among individuals, ranging greatly between 1 and 165 days. Because of the high sampling effort and similar return rates between individuals of different residency categories, our evidence is fairly compelling that shorter stays truly reflect shorter residencies and not detectability issues, predation, or mortality. Overall, the mean persistence duration was similar throughout the years, so residency dynamics at a species level may not be changing strongly with time. In the Gambia, Whitethroats were also observed to have different degrees of site persistence with 45% of captured individuals remaining in the area between two and 84 days, though most individuals were caught less than a month after ringing (King & Hutchinson, 2001). In Senegal, however, most individuals were on the passage (King & Hutchinson, 2001).

Different wintering strategies of individuals at the same site have also been recorded for other long‐distance migrants: 26% of Blackcaps *Sylvia atricapilla* in Spain (Belda et al., 2007) and 8% of Chiffchaffs in Portugal (Catry et al., 2003) were categorized as winter residents, while the rest were categorized as transients. This could have several explanations. First, individuals could have genetic differences due to parallel evolution of morphological and behavioral adaptations, making some individuals more inclined to lead either a nomadic or a resident lifestyle (Senar & Borras, 2004). We cannot, however, provide evidence for this, as we do not know whether individuals categorized as “passage” or “unknown” at our study site remained itinerant throughout the season, if they were in fact *en route* to a stationary nonbreeding site elsewhere, or if they died during the period. These genetic differences could also reflect individuals from different breeding populations, but this seems highly unlikely because individuals switched strategies across years, and Whitethroats seem to have a somewhat low migratory connectivity (Tapia‐Harris et al., 2022).

A second explanation could be due to habitat quality changes throughout the season. Whitethroats arrive at the study site at the end of the rainy season, between September and November, when vegetation is dense, and resources are abundant (Hulme & Cresswell, 2012; Nwaogu & Cresswell, 2016; Zwarts et al., 2009). As the season progresses, precipitation and crop cover are low or nonexistent, the occurrence of grazing, bushfires, and wood extraction increases (Hulme & Cresswell, 2012), and shrub cover, an important component of a Whitethroat's habitat, decreases (Tapia‐Harris & Cresswell, n.d.). The broad residency spectrum, from continual and variable movement to winter residency, could reflect a gradient in the predictability of food supplies (Belda et al., 2007; Newton, 2008). As habitats change, some individuals may decide to leave the area to find other more suitable habitats elsewhere, while others may risk staying.

Short‐term residencies, during both autumn and spring, may indicate the use of multiple important nonbreeding sites. Nigerian Whitethroats deployed with geolocators remained at the first stationary nonbreeding site in the Sahel before arriving at our study site in November (Tapia‐Harris et al., 2022). If some of these birds were then to be short‐term residents, individuals could have more than two important stationary nonbreeding sites. These results support the increasing evidence that the use of multiple wintering sites by Afro‐Palearctic migrants may be more common than previously thought (Blackburn & Cresswell, 2016; Burgess et al., 2020; Lemke et al., 2013; McKinnon et al., 2013).

Adult birds' site persistence was longer than first‐year birds likely due to their previous experience. An individual's first nonbreeding period is full of uncertainty, thus many first‐year birds probably arrive stochastically at breeding sites looking for suitable habitats (Cresswell, 2014). Most of these birds will need to explore the terrain and scout for resources, and some may ultimately remain for the entire nonbreeding season, while others will only remain for a portion of the season before moving to a second nonbreeding site. Still, others may immediately continue their search elsewhere, migrating further after only a brief stay.

### 4.3 Between‐years site fidelity

Many long‐distance migrants return to the same nonbreeding sites year after year both in the Nearctic‐Neotropical and Afro‐Palearctic systems (Blackburn & Cresswell, 2016; Moreau, 1969; Salewski et al., 2000). Here we found that a minimum of 19% of individuals returned from 1 year to the next, an intermediate return rate in comparison to other Palearctic migrants in Africa (Kelsey, 1989; Salewski et al., 2000; Thorup et al., 2019) and Whitethroats at their breeding grounds (0%–64%; da Prato & da Prato, 1983, 14.5%; Boddy, 1992). Not only did a significant proportion of individuals return the following year but individuals moved, on average, 300 m. These results suggest that many individuals have a high degree of between‐years site fidelity at a very small spatial scale, but these results are biased to individuals detected within our study plots and exclude those individuals that may have settled close by, just outside of our study site. Fidelity across years confers the same advantages as longer residency patterns, especially regarding knowledge of local and fluctuating food sources, competitor densities, and location of refuges, and this seems to secure and increase an individual's survival (Brown & Long, 2006; Piper, 2011).

Return rates were different among individuals from different age categories: first‐years had lower return rates than adults, at least from year 2 to year 3. This result further highlights the inexperience and stochastic nature of first‐year birds. Older birds, on the other hand, will tend to reuse nonbreeding sites, so becoming more site faithful over time (Cresswell, 2014).

### 4.4 Departure dates

Individuals did not seem to change their departure timing from year 1 to year 2, although further information over many years is needed to draw stronger conclusions. Timing of migration is of critical importance in migratory species and is key for securing fitness (Drent et al., 2003; Kokko, 1999). Departure from the nonbreeding grounds has been seen to correlate with arrival at breeding sites (Kristensen et al., 2013; Ouwehand & Both, 2017), though later departing individuals can migrate faster to compensate for lost time (Yohannes et al., 2009). As our study site is located in the southern part of the distribution and individuals have different breeding sites, the first individuals to depart are not necessarily the first to arrive at their respective breeding grounds (Tapia‐Harris et al., 2022).

Individuals at our study site showed relatively low repeatability departure values (*r =* .15) compared with other Afro‐Palearctic migrants (Both et al., 2016). Low repeatability fits well with the idea that individuals do not always remain for similar periods across years, and therefore, it is perhaps not surprising that some individuals left the area on different dates, but there are a few things to consider with these results. Firstly, repeatability indicates how consistently individuals differ from each other and is not necessarily a measure of individual repeatability across years (Conklin et al., 2013). Secondly, departure from our study site does not necessarily imply that individuals commenced spring departure; they could have moved to another nonbreeding site.

## 5 CONCLUSION

Our results are consistent with the serial residency hypothesis (Cresswell, 2014) but also suggest small modifications. Instead of individuals repeatedly settling at the same wintering sites during the same periods year after year, we hypothesize that individuals may vary their timing at the same sites depending on yearly conditions throughout the migratory routes, especially changes in sites where individuals remain stationary. In summary, results suggest that spatial fidelity is high and constant through years, but temporal use or temporal fidelity and site persistence may vary and a possible explanation for this might be variation in yearly and seasonal conditions and in habitat quality. In other words, timing is important: individuals revisit locations at very precise scales but do not necessarily repeat them at the same time. There is temporal flexibility but not spatial flexibility, except in the sense there is always an option to make a potentially dangerous further migration to an unknown area if conditions became untenable. But these hypotheses can only be tested fully when small birds such as Whitethroats can be tracked with nonarchival tags so that wintering locations regardless of site fidelity and long‐term survival can be seen.

## AUTHOR CONTRIBUTIONS

**Claudia Tapia‐Harris:** Conceptualization (equal); data curation (lead); formal analysis (lead); funding acquisition (supporting); investigation (lead); project administration (lead); resources (supporting); visualization (lead); writing – original draft (lead); writing – review and editing (equal). **Will Cresswell:** Conceptualization (equal); formal analysis (supporting); funding acquisition (lead); resources (lead); supervision (lead); writing – original draft (supporting); writing – review and editing (equal).

## CONFLICT OF INTEREST

The authors declare no competing interests.

## Figures and Tables

**FIGURE A1:**
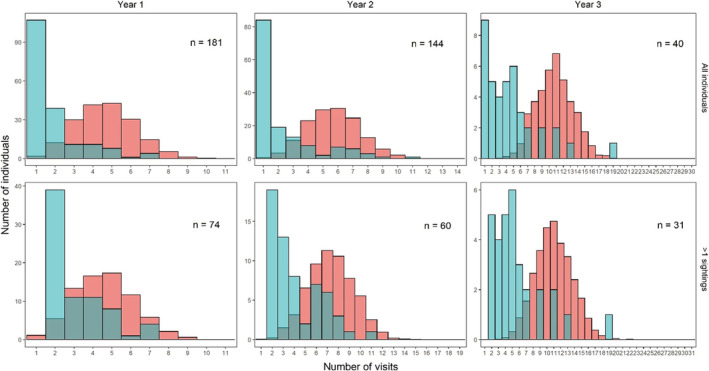
Expected (red) and observed (blue) frequencies of the number of individuals that were detected at the different number of visits. Graphs in the first row were obtained from data gathered from all individuals and graphs from the second row were obtained from data of individuals that were seen at least twice during the year. In all graphs, blue bars (observed data) and red bars (expected data) rarely overlap. We observed many more individuals than expected only once or twice if all were long‐term residents when the detectability rate was assumed to be 0.33.

## Data Availability

The research data underpinning this publication can be accessed at https://doi.org/10.17630/a8346f8b‐47cb‐42bf‐affc‐68b101dd859a.

## APPENDIX A Proving different residency categories

To provide evidence that individuals remained for different periods throughout the nonbreeding season at our study site—that individuals have different within‐winter residency strategies—we compared the observed frequencies of the number of visits individuals were detected each year, with that expected by chance assuming that all individuals were long‐term winter residents. To do this, we first calculated the number of individuals seen *per* year and the respective mean number of visits. We ran 50 iterations to obtain a representative mean of birds detected each visit, assuming that (1) birds were present in the area throughout the study, i.e., long‐term winter residents, and (2) that the detection probability *per* visit was .33 (see manuscript). We then compared these estimates with our observed data using a two‐sample *t* test. We expect that if our observed frequencies match those expected by chance, then all individuals are long‐term winter residents. If frequencies do not match, however, we assume that individuals have different duration residency periods. All years were analyzed separately. Individuals seen over multiple years were not excluded from any analyses.

We repeated this same analysis using data from individuals that were observed at least twice throughout the year to eliminate individuals that were likely to be simply passage birds. By doing this, we eliminate individuals that may have been passing by and detected by chance, and not necessarily utilizing resources from the area. If afterward we still observe differences in expected and observed frequencies, then we expect that not all individuals detected at our study site are long‐term winter residents.

We found that the expected frequencies of the number of visits that individuals were predicted to be detected at was statistically different from what was observed when analyzing both the data set with information from all individuals and the data set with individuals that were detected at least twice during the year (Figure A1; Table A1 and B1). These results are similar across years. With this, we confirm that not all birds seen at our study sites are long‐term winter residents.

**TABLE A1:** Statistical summary of the expected and observed number of visits at which individuals were detected during all 3 years and using two data sets: (1) “all data,” information from all detected individuals, and (2) “>1 sightings,” information from individuals detected at least twice during the year. All the expected mean visits were statistically different from the observed mean visits

Year	Data set	*n*	Expected mean visits	SE	Observed mean visits	SE	Results from *t* tests
1	All data	181	4.64	0.12	1.86	0.1	*t*_(360)_ = 18, *p* < .001***
1	>1 sightings	74	4.62	0.19	3.09	0.17	*t*_(146)_ = 6.1, *p* < .001***
2	All data	144	5.67	0.14	2.3	0.18	*t*_(286)_ = 14.9, *p* < .001***
2	>1 sightings	60	7.3	0.27	4.12	0.29	*t*_(118)_ = 8.1, *p* < .001***
3	All data	40	10.8	0.39	4.62	0.61	*t*_(78)_ = 8.6, *p* < .001***
3	>1 sightings	31	10.8	0.46	5.68	0.68	*t*_(60)_ = 6.3, *p* < .001***

*** Indicate highly significant values (p < .001)

## APPENDIX B Distances shifted between years

**TABLE B1:** Descriptive statistics of the distance (in m) shifted between years according to groups, previous age, sex, and previous residency. Group A = individuals detected in years 1 and 2, B = individuals detected in years 2 and 3, and C = individuals detected in years 1 and 3 but were not seen in year 2.

Variable		*n*	Min distance	Max distance	Mean distance	SE	Median distance
Group	A	28	6.3	2106.9	298	101	99.2
	B	21	7.8	2239.6	285	129	71.7
	C	5	80.1	942.7	273.1	167	120.1
Previous age	Adults	28	7.8	1790.8	203.8	73	81.3
	First‐years	19	6.3	2239.6	441.7	169	99.7
Sex	Females	20	6.3	1018.1	148.1	50	99.7
	Males	15	13	1790.8	241.7	125	71.7
Previous residency	Long‐term	28	6.3	2239.6	207	179	55.9
	Short‐term	6	71.7	288.1	130.3	32	106.8
	Passage	16	17.5	2106.9	481.4	178	132.5
